# How Much Protoporphyrin IX Must Be Activated to Obtain Full Efficacy of Methyl Aminolevulinate Photodynamic Therapy? Implication for Treatment Modifications

**DOI:** 10.3390/ph14040333

**Published:** 2021-04-06

**Authors:** Hans Christian Wulf, Ida M. Heerfordt, Peter Alshede Philipsen

**Affiliations:** Department of Dermatology, Copenhagen University Hospital Bispebjerg, 2400 Copenhagen NV, Denmark; ida.marie.heerfordt@regionh.dk (I.M.H.); peter.alshede.philipsen@regionh.dk (P.A.P.)

**Keywords:** actinic keratosis, illumination time, incubation time, photodynamic therapy, protoporphyrin IX

## Abstract

Photodynamic therapy (PDT) with methyl aminolevulinate (MAL) is a popular treatment for actinic keratoses (AK), and several PDT treatment modalities with similar cure rates are in use. The effect relies on the activation of protoporphyrin IX (PpIX) in premalignant cells. This study aimed to measure PpIX during each treatment modality to determine the minimal PpIX activation and shortest exposure time for optimal cure rate. In four different treatment modalities, we established the PpIX formation up to three hours after MAL application without illumination and measured the speed of PpIX photoactivation during 9 min of red light (37 J/cm^2^). The level of PpIX three hours after MAL application was set to 100 PpIX units. In comparison, 85 PpIX units were formed during daylight PDT, 57 PpIX units during pulse PDT, and 52 PpIX units without any curettage prior to MAL. The activation of 50 PpIX units should, therefore, be enough to obtain a full effect on AK. Further, red light illumination may be shortened from 9 min to 1–2 min. The results indicate that PDT can be performed successfully with half the illumination time used in daylight PDT today and with one fourth of the illumination time used in classical PDT.

## 1. Introduction

Classic photodynamic therapy (PDT) is associated with pain during illumination but also with great inter-patient variation in pain severity when treating actinic keratosis (AK). Approximately 10% of all patients are unable to complete the illumination due to pain [1]. Acute pain sets in almost immediately when switching on the lamp and, due to pain, illumination is frequently disrupted after just a few minutes [2]. Despite this, anecdotes of unchanged treatment efficacy persist.

The classic method involves curettage, application of methyl aminolevulinate (MAL) cream (160 mg/g), and approximately 9 min of illumination with red diode light (Aktilite^®^ 37 J/cm^2^) three hours after MAL application [3,4].

With the intention of reducing pain and inflammation, several alternative ways of performing PDT of AK on the face and scalp have been developed, all producing similar cure rates. Most of these modalities build on replacing the short-time red light exposure with continuous activation of protoporphyrin IX (PpIX) during its formation by long-term, low-intensity light exposure. Any light absorbable in PpIX can be used, such as daylight or lamp-emitted artificial daylight [5].

Classic daylight PDT involves curettage, MAL application, continuous daylight illumination for 2 h, starting 30 min after application and ending 2.5 h after application [6].

Pulse PDT [7] involves curettage, MAL application, and removal of cream residue after 30 min. Illumination is performed with red diode light 37 J/cm^2^ three hours after application; alternatively, continuous daylight exposure for 2 h, starting 30 min after application and ending 2.5 h after application, may be used [8].

Daylight PDT without curettage [9] involves MAL application to the face and scalp without pre-treatment. The continuous daylight illumination begins after 1 h and ends 3 h after MAL application.

**Figure 1:**
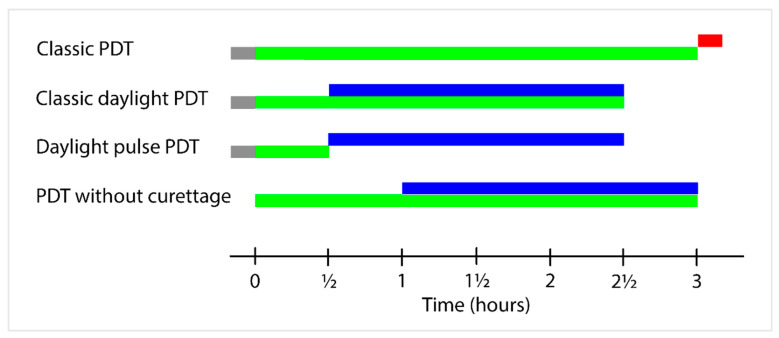
Time course of the single components in the four different photodynamic therapy (PDT) modalities. In most cases, skin is curettaged before MAL application (**grey color**). Methyl aminolevulinate (MAL) cream is applied at time 0. MAL incubation time (**green color**). Duration of illumination with daylight (**blue color**) or red diode light (Aktilite^®^ 68 mW/cm^2^ (**red color**)).

The time course of the single components in the four PDT modalities is illustrated in Figure 1.

The efficacy of PDT builds on the formation of PpIX in the mitochondria, and its activation by appropriate light wavelengths causes cell death. When the PpIX level is kept low, pain and inflammation are lessened [2,10].

It is not clearly established how much PpIX needs to be formed and activated for optimal efficacy. As all presented methods are reported to have the same cure rate on AK of the face and scalp, the intention of this paper is to evaluate how much PpIX is formed and activated when performing classical PDT with short-term illumination 3 h after MAL application, or long-term continuous daylight illumination ending 2.5 h after MAL application. Thereby it is estimated how much shorter the MAL incubation time during PDT treatments could be. Additionally, we aim to establish whether shorter illumination time in classic PDT is likely to give a full effect when treating AK of the face and scalp.

## 2. Results

### 2.1. PpIX Formation

**Figure 2:**
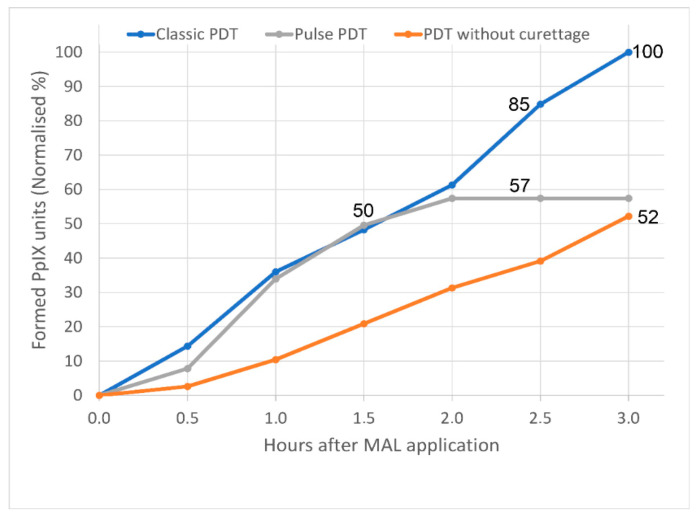
Time-dependent protoporphyrin IX (PpIX) formation without light exposure, when using different photodynamic therapy (PDT) treatment modalities. PpIX formation was measured by surface fluorescence. The PpIX units available for photoactivation are shown for classic PDT (blue line), daylight PDT (blue line up to 2.5 h), daylight pulse PDT (grey line), and when no curettage was performed before methyl aminolevulinate (MAL) application (orange line). PpIX formed 3 h after curettage and MAL application has been normalized to 100%. The activatable PpIX units in the used treatment modalities are shown at 2.5 h and 3 h after MAL application. The PpIX units formed after the suggested shorter treatment time of 1½ h is shown to be 50 PpIX units (50% of the PpIX formed in classic PDT).

PpIX is an endogenous fluorophore and PpIX formation was measured by surface fluorescence. All data on PpIX formation representing the four different treatment modalities are presented in Figure 2.

(I) The amount of PpIX after 3 h in the +Cur3hourMAL group (see Methods) was normalized to 100 PpIX units, being the highest amount of PpIX measured in the study. One hundred PpIX units are the amount of PpIX available for photoactivation 3 h after MAL application before illumination with red light in classic PDT.
(II) After 2.5 h, 85 PpIX units were formed in the +Cur3hourMAL group, representing the amount of PpIX available for photoactivation during classic daylight PDT.
(III) After 2.5 h, 57 PpIX units were formed in the +Cur30MinMAL group, representing the amount of PpIX available for photoactivation during daylight pulse PDT.
(IV) When no curettage is performed, 3 h after MAL application, 52 PpIX units were formed in the -Cur3hourMAL group, representing the amount of PpIX available for photoactivation during PDT without curettage.

AK cure rate is identical in all four described treatment modalities.

### 2.2. PpIX Photoactivation

**Figure 3:**
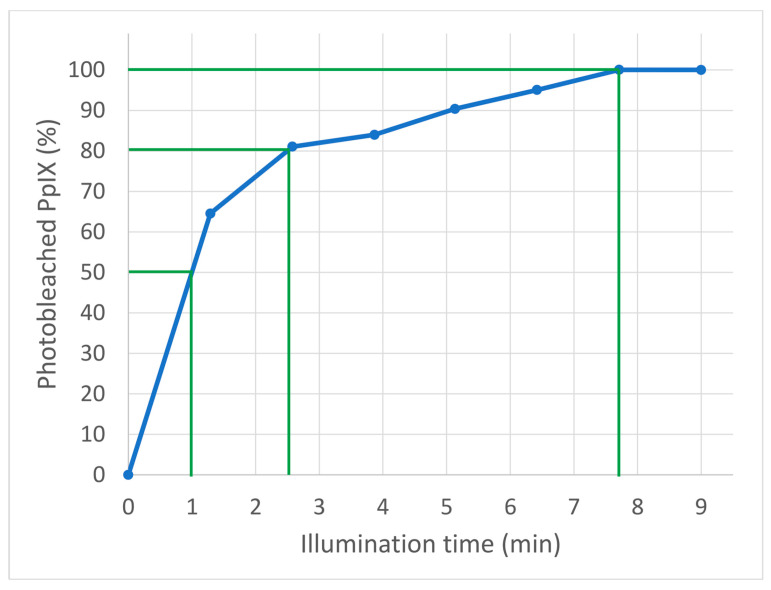
The curve shows the amount of photobleached protoporphyrin IX (PpIX) during 9 min of illumination by red diode light (Aktilite^®^ 68 mW/cm^2^) as used in classic photodynamic therapy. Illumination was performed after 3 h of methyl aminolevulinate incubation. Decrease in PpIX fluorescence (photobleaching) was interpreted as photoactivated PpIX.

During photoactivation, PpIX is converted into a nonfluorescent product. In classic PDT, approximately 9 min of illumination with red diode light (Aktilite^®^ 68 mW/cm^2^) is performed after 3 h of MAL incubation. The PpIX level just before the start of illumination was set at 100%. All 100 PpIX units were photoactivated after 7.75 min, 80 PpIX units were photoactivated after 2.5 min, and 50 PpIX units were photoactivated after 1 min of illumination. All data on PpIX photoactivation are shown in Figure 3.

## 3. Discussion

A certain amount of PpIX must be formed and activated to achieve optimal efficacy and the light intensity must be high enough to activate all formed PpXI [5,11]. Activation speed has been measured and illustrated in Figure 3. This allows us to estimate how many units of PpIX are activated per time unit. There is no indication, however, of activation speed being of importance as lasers, daylight, and classic red light with very different illumination times may all be used. This indicates that the activation curve is different when using lamps with different light intensities and different light spectra.

It has previously been shown that increasing the amount of PpIX to a higher level than that of classic PDT does not increase treatment efficacy on AK of the hands [10]. Further, the pain associated with PDT is considerably worse when more PpIX is activated during illumination, or if the daylight exposure time is prolonged, which also results in more PpIX activation [6]. Therefore, a sensible course could be reducing the illumination time with red light 3 h after MAL application to last just long enough to activate the necessary amount of PpIX. Fernández-Guarino et al. [12] halved the illumination time and still obtained the full treatment effect on AK. We have previously determined the minimal total PpIX weighted daylight dose for full efficacy to be 4–8 J/cm^2^ [6,11]. A similar light dose is obtained after 1–2 min of illumination with 68 mW/cm^2^, indicating that 2 min of illumination should be acceptable (Figure 3). This is less than one-fourth of the recommended time.

It is well known that the concentration of PpIX becomes higher when PDT is performed on the face and scalp, compared to other body locations, and illumination length may depend on the location of AK. Nevertheless, many studies have been performed with the purpose of increasing PpIX formation. Such attempts could be irrelevant, unless the distribution of PpIX in the skin is more optimal, or goes deeper into dermis, which might be of advantage in some cases [13].

The full obtainable effect on AK is achieved by the classic PDT approach. This is also the case when performing continuous activation with daylight PDT, even though only 85% of PpIX is activated compared to the amount activated in classic PDT. The full effect on AK of the face and scalp is also observed after activation of 57–52 PpIX units, as in pulse PDT and PDT without curettage [8,9]. Thus, continuous activation of 50 PpIX units specifically located intracellularly may be close to the minimum level of PpIX activation needed to obtain the full effect of the PDT treatment, but it is possible that the necessary PpIX activation may be even lower.

In pulse PDT, the amount of activatable PpIX is stable, between 2 and 3 h after MAL application (Figure 2), and red-light illumination 2 h after MAL or daylight up to 2 h after MAL application should be effective. As seen in Figure 2, the treatment may be shortened even more, as 50 PpIX units are already found 1.5 h after MAL application. This will further minimize the side effects from light exposure after the end of treatment. MAL in the skin is not removed or metabolized chemically by exposure to light and PpIX is formed while MAL is present, also after the end of treatment. Compared to the other modalities, the advantage of pulse PDT is the presence of very little residual MAL at the end of treatment. This lessens the risk of post-treatment inflammation due to random daylight exposure.

PDT without curettage still necessitates illumination up to 3 h after MAL application when 52 PpIX units are activated. The advantages of this modality are less pain and inflammation, and the avoidance of bleeding following pre-treatment curettage, which may present a problem in patients treated with anticoagulants. MAL provokes oozing and bleeding when applied to curettaged skin, which may necessitate reapplication of cream. When curettage is omitted, these problems are avoided.

Our calculations depend on the correctness of the PpIX formation curve in classic PDT as the other results are related to this. It is likely that this curve is credible as it is identical to the PpIX formation curve in pulse PDT for the first 2 h after MAL application (Figure 2), and to previous measurements (by use of a different technique) performed by Wiegell et al. [14,15].

The effectiveness of each PpIX unit depends on the high specificity of the drug to mainly target diseased cells. Most likely, the PpIX formed in the mitochondria must remain there for the PpIX units to have the greatest effect. This is best achieved by using continuous activation by light in order to activate every PpIX molecule as soon as it is formed in the mitochondria. Daylight PDT was developed for this purpose. Additionally, pulse PDT was developed to reduce PpIX formation after the end of treatment. When the PpIX formation time is prolonged before the start of illumination, PpIX leaks to other cell components, as well as extracellularly, resulting in excessive inflammation and possibly rendering each PpIX unit less effective. Treatment with MAL with a short incubation time represents a way to keep the PpIX in the mitochondria of the abnormal cells, thereby lessening the pain and inflammation while upholding efficacy. This may be obtained by use of the data presented here.

Earlier attempts to reduce treatment time were not based on a clear pharmacologic rationale. In 2008, Braathen et al. [16] used MAL cream (160 mg/g) and compared the cure rate of AK when illuminating 1 h after MAL application with red light at 75 J/cm^2^ (Curelight^®^, PhotoCure, Norway) to the cure rate of AK with illumination 3 h after MAL application by the same light source (classic PDT). Compared to illumination after 1 h, a moderately better cure rate was obtained with the classic method, indicating that one hour of PpIX formation is too short. If Figure 2 is applied, only around 35 PpIX units are formed after 1 h. The authors also indicate that debridement before MAL improves efficacy in both modalities.

In an uncontrolled study by von Dobbeler et al. [17], a full effect was obtained by use of a nanoemulsion of 5-aminolevulinic acid (ALA) (Ameluz^®^ 78 mg/g), after pre-treatment with curettage, degreasing, and ALA on the skin for 1 h, after which the skin was illuminated with white LED for 1 h (20 J/cm^2^). If the result is comparable to the results obtained by MAL, we might expect an activation of around 60 PpIX units after two hours (Figure 2). As ALA typically forms PpIX faster than MAL, even more PpIX may have been activated [18]. However, after ALA, more PpIX may be found in normal skin structures than when using MAL and this extra PpIX may not result in improved efficacy. As the ALA treatment was effective in this uncontrolled study on AK in the face and scalp, it indicates that 60% activation of PpIX is a rather safe level when enough light is present to activate all formed PpIX within the time range [11].

On the basis that activation of 50 PpIX units is sufficient to achieve the full effect of the treatment, the modalities used in the face and scalp may be translated into one of the following procedures:

Classic PDT including curettage, MAL application, followed by illumination for 9 min with Aktilite^®^ 68 mW/cm^2^, 37 J/cm^2^, 1.5 h after MAL application.

Classic PDT including curettage, MAL application, followed by illumination for 2 min with Aktilite^®^ 68 mW/cm^2^, in total 8 J/cm^2^, 3 h after MAL.

Classic daylight PDT including curettage, MAL application and continuous activation for 1 h of daylight, starting 30 min after application.

Pulse PDT including curettage followed by MAL application, removal of cream residue after 30 min, and illumination for 9 min with Aktilite^®^ 68 mW/cm^2^, 37 J/cm^2^, 1.5 h after MAL application.

Daylight pulse PDT including curettage, followed by MAL application, removal of cream residue after 30 min, and continuous illumination with daylight for 1 h, starting 30 min after MAL application.

It is unknown whether the shortened time of exposure to MAL and shortened time of illumination with red diode light can be combined. However, it is likely that illumination with red light for 9 min is still necessary when only 50 PpIX units are activated (after 1.5 h) if the activation speed is still logarithmic (Figure 3). Using a different measuring technique, Ericson et al. [19] have shown similar photobleaching as in Figure 3 and a relative independence of light intensity and light spectrum. This issue needs further investigation.

The efficacy of these radical changes in protocol needs testing by clinical trials before translation into clinical praxis.

## 4. Materials and Methods

### 4.1. PpIX Measurements

PpIX is an endogenous fluorophore emitting red light when excited by blue light [3]. PpIX was measured noninvasively by surface fluorescence using a handheld photometer (FluoDerm, Dia Medico ApS, Gentofte, Denmark) [20,21]. The photometer illuminates the skin with blue light (400–420 nm), matching the Soret band of PpIX at 407 nm. By measuring excitation wavelengths at 610–700 nm, the corresponding red PpIX fluorescence was detected. By this method, the PpIX concentration in the epidermis was roughly estimated [22].

### 4.2. PpIX Formation

Background fluorescence was measured before MAL application. Subsequently, fluorescence was measured during MAL incubation at different time points. Increase in measured fluorescence was presented as PpIX formation. Data on PpIX formation originate partly from a previous published study [23], supplemented with new data on the development of PpIX in 5 individuals older than 55 years of age during classic PDT of AK on the face and scalp.

In this study, we introduce the following definitions: −Cur3hourMAL represents PpIX formation during PDT without curettage [9], +Cur30MinMAL represents PpIX formation during pulse PDT [8], and +Cur3hourMAL represents PpIX formation during classic PDT.

During routine classic PDT treatment of AKs at the Department of Dermatology, Bispebjerg Hospital, we measured (i) PpIX formation every 30 min after curettage during 3 h of MAL incubation (+Cur3hourMAL) before illumination without interfering with the planned standard treatment. Further, we extracted data on PpIX formation every 30 min for 3 h: (ii) during MAL incubation without prior curettage (−Cur3hourMAL) and (iii) 30 min MAL incubation after curettage (+Cur30MinMAL) from a previous study [23]. All PpIX data were measured without light exposure and the measured PpIX represents the PpIX available for activation during PDT. All measurements in the different settings were performed by the same investigator (I.M.H.) and the highest amounts of PpIX measured were normalized to 100 PpIX units.

### 4.3. PpIX Photoactivation

All data on PpIX photoactivation were extracted from a previous study [24]. During photoactivation, PpIX is converted into a nonfluorescent photobleached product. PpIX fluorescence was measured after 3 h of MAL incubation just before illumination start and 7 times during illumination. As in classical PDT, the skin was illuminated by red diode light (Aktilite^®^ 68 mW/cm^2^) for 9 min. Decrease in PpIX fluorescence (photobleaching) was interpreted as photoactivated PpIX.

### 4.4. Statistics

All reported PpIX values were mean PpIX values.

## 5. Conclusions

In conclusion, most PDT treatment modalities can probably be performed after a considerably shorter MAL exposure time or a shorter illumination time without losing efficacy. When performing classic PDT, the illumination time can probably be shortened from around 9 min to a few minutes without losing efficacy. This indicates that cessation of illumination after a few minutes due to pain may not constitute a lack of efficacy in classic PDT.

## Data Availability

The data presented in this study are available on request from the corresponding author. The data presented in this study are available in this article.
